# Indirect Effects of Glucagon-Like Peptide-1 Receptor Agonist Exendin-4 on the Peripheral Circadian Clocks in Mice

**DOI:** 10.1371/journal.pone.0081119

**Published:** 2013-11-15

**Authors:** Hitoshi Ando, Kentarou Ushijima, Akio Fujimura

**Affiliations:** Division of Clinical Pharmacology, Department of Pharmacology, School of Medicine, Jichi Medical University, Shimotsuke, Tochigi, Japan; McGill University, Canada

## Abstract

Circadian clocks in peripheral tissues are powerfully entrained by feeding. The mechanisms underlying this food entrainment remain unclear, although various humoral and neural factors have been reported to affect peripheral clocks. Because glucagon-like peptide-1 (GLP-1), which is rapidly secreted in response to food ingestion, influences multiple humoral and neural signaling pathways, we suggest that GLP-1 plays a role in the food entrainment of peripheral clocks. To test this, we compared the effects of exendin-4, a GLP-1 receptor agonist, on mRNA expression of the clock genes (*Clock*, *Bmal1*, *Nr1d1*, *Per1*, *Per2*, and *Cry1*) with those of refeeding. In addition, we investigated whether exendin-4 could affect the rhythms of the peripheral clocks. In male C57BL/6J mice, although refeeding rapidly (within 2 h) altered mRNA levels of *Per1* and *Per2* in the liver and that of *Per1* in adipose tissue, a single i.p. injection of exendin-4 did not cause such changes. However, unlike the GLP-1 receptor antagonist exendin-(9–39), exendin-4 significantly influenced *Per1* mRNA levels in the liver at 12 h after injection. Moreover, pretreatment with exendin-4 affected the rapid-feeding-induced change in *Per1* not only in the liver, but also in adipose tissue, without effect on food intake. Furthermore, during light-phase restricted feeding, repeated dosing of exendin-4 at the beginning of the dark phase profoundly influenced both the food intake and daily rhythms of clock gene expression in peripheral tissues. Thus, these results suggest that exendin-4 modulates peripheral clocks via multiple mechanisms different from those of refeeding.

## Introduction

Circadian clocks, which are composed of transcriptional/translational feedback loops involving a set of clock genes, represent an adaptation to daily 24-h changes in the environment and enable organisms to maintain physiological homeostasis [1]. Recent studies have indicated that disrupted circadian clocks are associated with the pathophysiology of metabolic diseases. In humans, genetic variation in the clock genes is reportedly linked to susceptibility to metabolic disorders, including obesity, metabolic syndrome, type 2 diabetes, and hypertension [2]–[5]. Additionally, we have shown that mRNA levels of the clock genes in peripheral leucocytes are associated with fasting plasma glucose concentrations and the degree of obesity in healthy males [6], and that their expression rhythms are dampened in patients with type 2 diabetes [7]. Similarly, in mice, homozygous mutation in the *Clock* gene leads to the development of metabolic syndrome [8], and rhythmic mRNA expression of clock genes is blunted in the liver and adipose tissue of genetically obese diabetic mice [9], [10]. Remarkably, peripheral tissue-specific *Clock* mutant mice also develop glucose intolerance [11], and liver-specific [12] and pancreas-specific *Bmal1* knockout mice [13] exhibit disrupted hepatic glucose production and reduced insulin secretion, respectively. Collectively, these findings suggest the possibility that circadian clocks, especially those in peripheral tissues (peripheral clocks), may be novel targets for the prevention and/or treatment of metabolic diseases.

Peripheral clocks are synchronized by the central clock residing in the hypothalamic suprachiasmatic nucleus (SCN) through probably multiple humoral and neural signals [1]. Therefore, peripheral clocks are entrained also by feeding, which affects the concentrations of various nutrients/hormones and nervous system activities, and the effect of feeding is greater than by that of light, which is the time cue for resetting the central clock [1], [14]. Because the mechanisms underlying this food entrainment remain unclear, examining them may be useful in developing methods to control the peripheral clocks.

Glucagon-like peptide-1 (GLP-1) is an incretin hormone secreted by L cells located mainly in the distal small intestine and colon [15]. The plasma concentration of GLP-1 is elevated rapidly, within a few minutes, after oral glucose administration in rodents and humans [16]. Other nutrients (fat and amino acids) also induce biphasic GLP-1 release, with an early phase, beginning within minutes, and a second phase lasting up to 120 min or longer [17]. GLP-1 causes its postprandial glucose-lowering properties mainly through stimulating insulin secretion and inhibiting glucagon release [15], [16], [18]. In addition to these actions on pancreatic islets, GLP-1 regulates hepatic glucose uptake and production and gastric emptying and acid secretion at least partly through the vagus nerve [15], [18]. Thus, GLP-1 affects multiple humoral and neural signaling pathways in response to food ingestion. Considering these properties, we suggest that GLP-1 may play a role in the food entrainment of the peripheral clocks. To test this, we compared in mice the effects of exendin-4, a GLP-1 receptor agonist, on the mRNA expression of the clock genes to those of refeeding. Moreover, we investigated whether exendin-4 could affect the rhythms of the peripheral clocks.

## Materials and Methods

### Ethics statement

The protocol for the study was approved by the Institutional Animal Experiment Committee of Jichi Medical University (Shimotsuke, Japan; Permission numbers: 1140, 12184, and 13168). All animal procedures were performed in accordance with the Institutional Regulation for Animal Experiments and Fundamental Guideline for Proper Conduct of Animal Experiment and Related Activities in Academic Research Institutions under the jurisdiction of the Ministry of Education, Culture, Sports, Science and Technology of Japan. All efforts were made to minimize animal suffering.

### Mice and treatments

Male C57BL/6J mice (Charles River Japan, Yokohama, Japan) were obtained at 8 weeks of age and maintained under specific pathogen-free conditions and controlled temperature and humidity with a 12/12-h light/dark (07:00–19:00 h/19:00–07:00 h) cycle. Mice were housed individually (in Experiments 1, 2, 3 and 5) or group-housed (4–5 animals/cage; in Experiment 4), and fed a regular diet (CE-2; CLEA Japan, Tokyo, Japan) and water *ad libitum*.

**Figure 1:**
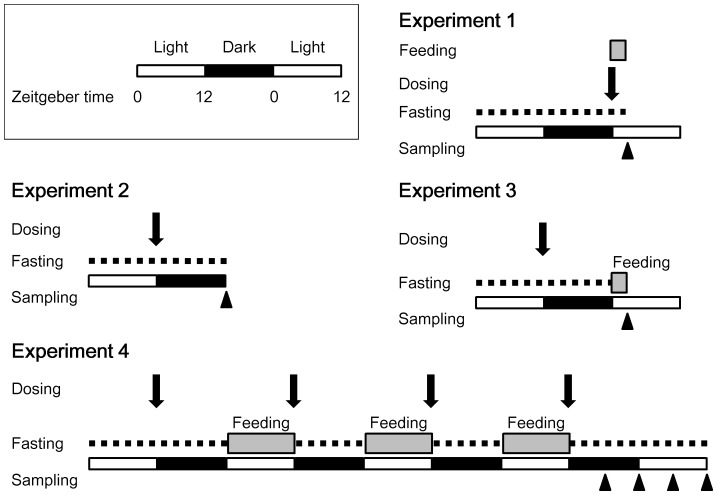
Experimental protocols. Male C57BL/6J mice were maintained under a 12-h light (white bars)/12-h dark (black bars) cycle. Mice were fasted (dashed lines), and thereafter, i.p. administered the study solution (arrows; exendin-4, exendin-(9–39) or vehicle) and/or fed (gray bars). Arrowheads indicate sampling time points.

Ten-week-old mice were administered saline i.p. containing 0.02% mouse serum albumin (Sigma-Aldrich, St. Louis, MO) with or without 10 nmol/kg exendin-4 (Sigma-Aldrich), as described below (Fig. 1). We selected the dose of 10 nmol/kg because repeated i.p. injections of this dosage to diabetic mice were reported to improve not only glycemic control [19], but also the peripheral nerve function [20]. The other groups of mice were injected i.p. with the same dose of exendin-(9–39) (Sigma-Aldrich), a GLP-1 receptor antagonist. This analog is often used to examine the physiologic consequences of loss of GLP-1 receptor signaling [15], and the dose used in this study has been reported to inhibit the actions of endogenous GLP-1 [21].

### Experiments

We carried out five experiments (Fig. 1 and S1A). Zeitgeber time (ZT) was used to describe the experimental time, with ZT 0 defined as lights on and ZT 12 as lights off.

#### Experiment 1 (Effects of exendin-4 on the circadian clocks at 2 h after administration)

Ten-week-old mice (*n* = 21) were divided into four groups and fasted from ZT 0 for 24 h. The regular diet was fed only to the refed group (*n* = 6) from ZT 0. The other groups were administered exendin-4 (*n* = 5), exendin-(9–39) (*n* = 4), or vehicle (*n* = 6) at ZT 0. At 2 h later (ZT 2), the mice were sacrificed to obtain liver, epididymal fat, and SCN samples. The SCN sample was dissected from the 0.5-mm coronal brain slice containing the optic tract, using Harris Uni-Core (0.5 mm inner diameter; Ted Pella, Redding, CA).

#### Experiment 2 (Effects of exendin-4 on the circadian clocks at 12 h after administration)

Fifteen mice were divided into three groups (*n* = 5 each) and fasted from ZT 0 for 24 h. At 12 h after the beginning of fasting (ZT 12), animals were given exendin-4, exendin-(9–39), or vehicle. The mice were sacrificed to obtain liver, epididymal fat, and SCN samples at ZT 0.

#### Experiment 3 (Effects of pretreatment with exendin-4 on refeeding-induced changes in the circadian clocks)

Sixteen mice were divided into three groups and fasted from ZT 0 for 24 h. The mice were given exendin-4 (*n* = 5), exendin-(9–39) (*n* = 5), or vehicle (*n* = 6) at 12 h after the beginning of fasting (ZT 12), and thereafter refed the regular diet from ZT 0. We found that all of the mice started eating within several minutes. Sampling of liver and epididymal fat was conducted at ZT 2.

#### Experiment 4 (Effects of repeated administration of exendin-4 on the circadian clocks)

Forty-six mice were divided into three groups, fasted from ZT 0 for 24 h, and thereafter fed the regular diet only during the light phase (ZT 0–12) for 3 days. Animals were administered exendin-4 (*n* = 12), exendin-(9–39) (*n* = 13), or vehicle (*n* = 21) once daily at ZT 12 for 4 days. Sampling of the peripheral tissues was performed at ZT 18, 0, 6, and 12.

#### Experiment 5 (Effects of exendin-4 on blood glucose and serum insulin concentrations)

Twenty mice were divided into two groups and fasted from ZT 0 for 24 h. The mice were given exendin-4 (*n* = 10) or vehicle (*n* = 10) at 12 h after the beginning of fasting (ZT 12), and thereafter refed the regular diet from ZT 0. Tail blood glucose levels were measured using a Glutest Ace R (Sanwa Kagaku Kenkyusyo, Nagoya, Japan) before (ZT 0) and at 12 (ZT 12) and 24 h (ZT 0) after the beginning of fasting and at 2 h after refeeding (ZT 2). At ZT 0 or 2 of the second day, the mice were sacrificed by cervical dislocation, and cardiac puncture was performed to obtain blood samples for insulin determination. Serum insulin concentrations were measured using a Mouse Insulin ELISA KIT RTU (Shibayagi, Shibukawa, Japan). Because one mouse in the exendin-4 group died during the experiment due to a technical reason, the data of the remaining 19 mice were analyzed.

### RNA extraction and real-time quantitative PCR

Total RNA isolated from the peripheral tissues, using an RNeasy Mini Kit or an RNeasy Lipid Tissue Mini Kit (Qiagen, Valencia, CA), was reverse-transcribed using a PrimeScript RT reagent Kit (Takara Bio, Otsu, Japan). SCN samples were directly reverse-transcribed using a TaqMan Fast Cells-to-Ct Kit (Life Technologies, Carlsbad, CA). Gene expression was analyzed by real-time quantitative PCR, performed using the Applied Biosystems StepOnePlus Real-Time PCR System (Life Technologies). Specific sets of primers and TaqMan probes (TaqMan Gene Expression Assays) were obtained from Life Technologies. To control for variation in the amount of cDNA available for PCR in the different samples, expression of target sequences was normalized to that of an endogenous control, ribosomal protein, large, P0 (*Rplp0*). Because we found that the Ct values of *Rplp0* were not influenced by either the treatments or sampling time in this study (Fig. S2), we used this gene as an endogenous control. The GenBank accession numbers, assay ID, and target exons, respectively, were: NM_007715.5, Mm00455950_m1, and 15–16 (*Clock*); NM_007489.3, Mm00500226_m1, and 11–12 (*Bmal1*); NM_145434.3, Mm00520708_m1, and 1–2 (*Nr1d1*); NM_011065.4, Mm00501813_m1, and 18–19 (*Per1*); NM_011066.3, Mm00478113_m1, and 19–20 (*Per2*); NM_007771.3, Mm00514392_m1, and 1–2 (*Cry1*); and NM_007475.5, Mm00725448_s1, and 7–7 (*Rplp0*). Data were analyzed using the comparative threshold cycle method.

### Statistical analysis

Data, presented as means ± SD, were analyzed using ANOVA followed by Bonferroni *post hoc* testing, or by Student's *t* tests. The calculations were performed using the SPSS software (ver. 16.0 J for Windows; Japan IBM, Tokyo). Daily rhythmicity was evaluated by the cosinor method, using an online open access program described by Refinetti *et al*. [22]. A *P* value<0.05 was considered to indicate statistical significance.

## Results

### Refeeding, but not exendin-4, rapidly affected peripheral clocks

**Figure 2:**
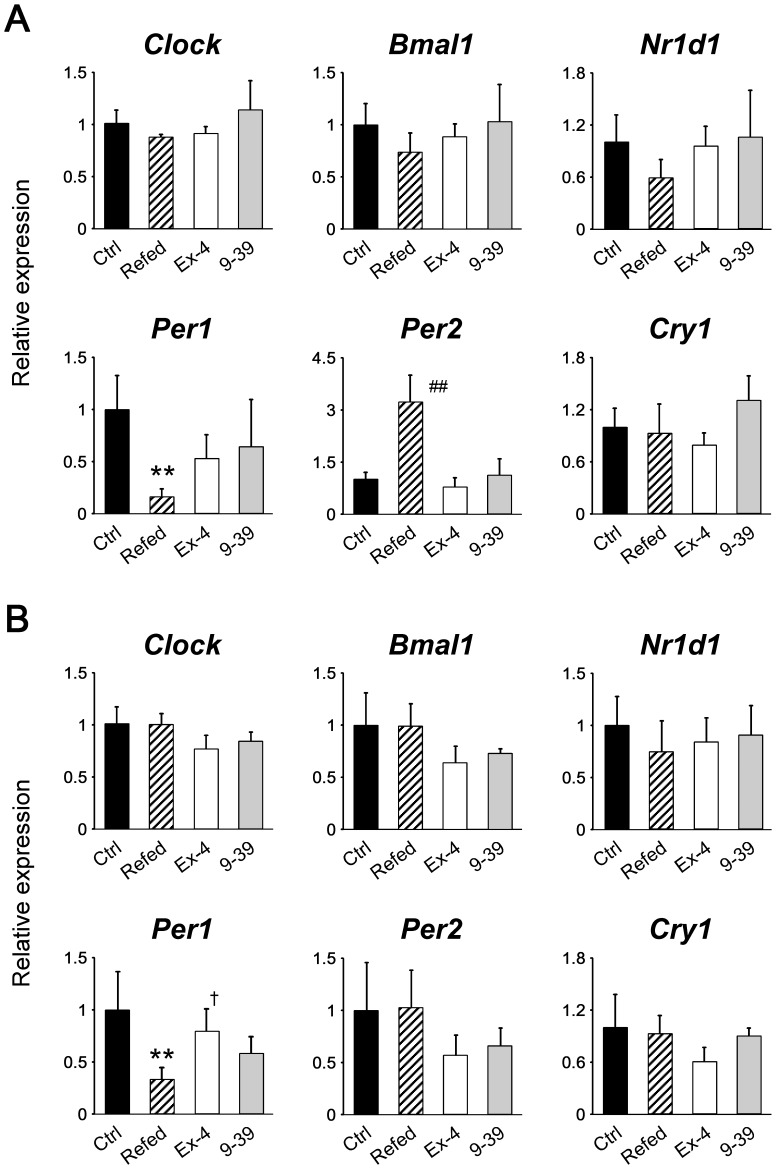
Effects of refeeding, exendin-4, and exendin-(9–39) on mRNA levels of the clock genes in the liver (A) and adipose tissue (B) at 2 h after the procedures (Experiment 1). Samples were obtained from control (Ctrl; black bars, *n* = 6) and refed (striped bars, *n* = 6) groups, and mice treated with exendin-4 (Ex-4; white bars, *n* = 5) and with exendin-(9–39) (9–39; gray bars, *n* = 4). The mean value of the control group was set to 1, and data represent means + SD. **, *P*<0.01 *vs*. the control group; ^##^, *P*<0.01 *vs*. all other groups; ^†^, *P*<0.05 *vs*. the refed group (by one-way ANOVA followed by Bonferroni *post hoc* testing).

Recently, it has been reported that refeeding induces, within 2 h, an increase in *Per2* mRNA expression and a decrease in *Nr1d1* mRNA expression in the livers of mice [23], [24]. Consistent with this, refeeding (in Experiment 1) increased *Per2* mRNA levels significantly and tended to decrease *Nr1d1* levels in the liver after 2 h (Fig. 2A). Additionally, we found that refeeding also decreased *Per1* levels significantly. Regarding adipose tissue, levels of only *Per1* were significantly influenced by refeeding among the clock genes examined (Fig. 2B). In contrast, neither exendin-4 nor exendin-(9–39) induced any change in mRNA levels of the clock genes examined in either peripheral tissue (Fig. 2A, 2B). Like refeeding, which is known not to influence the central clock [25], these analogs did not affect the circadian clock in the SCN (Fig. S3).

### Effect of exendin-4 on the hepatic clock becomes obvious at 12 h after administration

**Figure 3:**
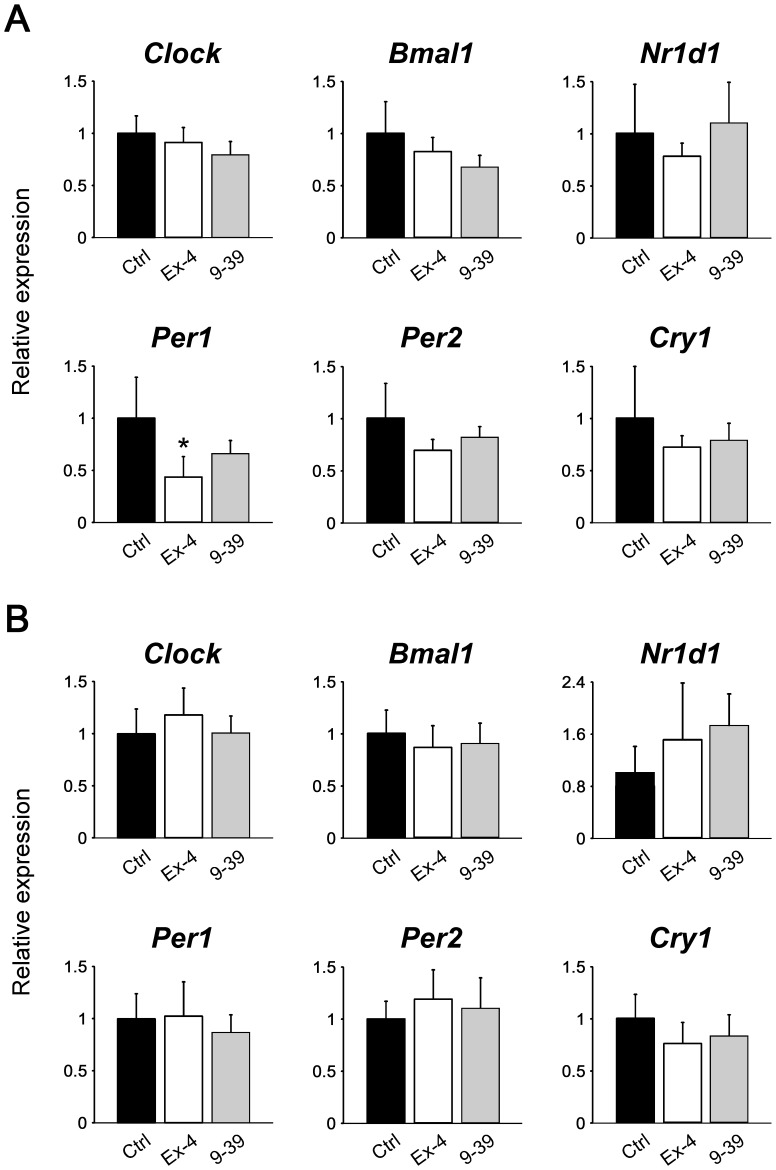
Effects of exendin-4 and exendin-(9–39) on mRNA levels of the clock genes in the liver (A) and adipose tissue (B) at 12 h after administration (Experiment 2). Samples were obtained from mice treated with vehicle (Ctrl; black bars, n = 5), exendin-4 (Ex-4; white bars, n = 5), and exendin-(9–39) (9–39; gray bars, n = 4). The mean value of control group was set to 1, and data represent means + SD. *, *P*<0.05 *vs*. the control group (by one-way ANOVA followed by Bonferroni *post hoc* testing).

We next examined whether exendin-4 affects the clocks in a delayed manner (Experiment 2). As shown in Figure 3A, exendin-4, but not exendin-(9–39), significantly decreased *Per1* levels in the liver at 12 h after injection (ZT 0). No effects of both analogs were observed in the adipose tissue (Fig. 3B) and SCN (Fig. S4). The treatment with exendin-4 (in Experiment 5) did not influence fasting blood glucose levels at ZT 0 (60.3±17.5 mg/dl in the exendin-4 group *vs*. 60.8±11.0 mg/dl in the control group; *P* = 0.94 by Student's *t* test; Fig. S1B). In addition, serum insulin concentrations at ZT 0 were below the detection limit (100 pg/ml) in all mice examined (both exendin-4 and control groups; *n* = 5 in each group).

### Exendin-4 affects refeeding-induced changes in peripheral clocks

**Figure 4:**
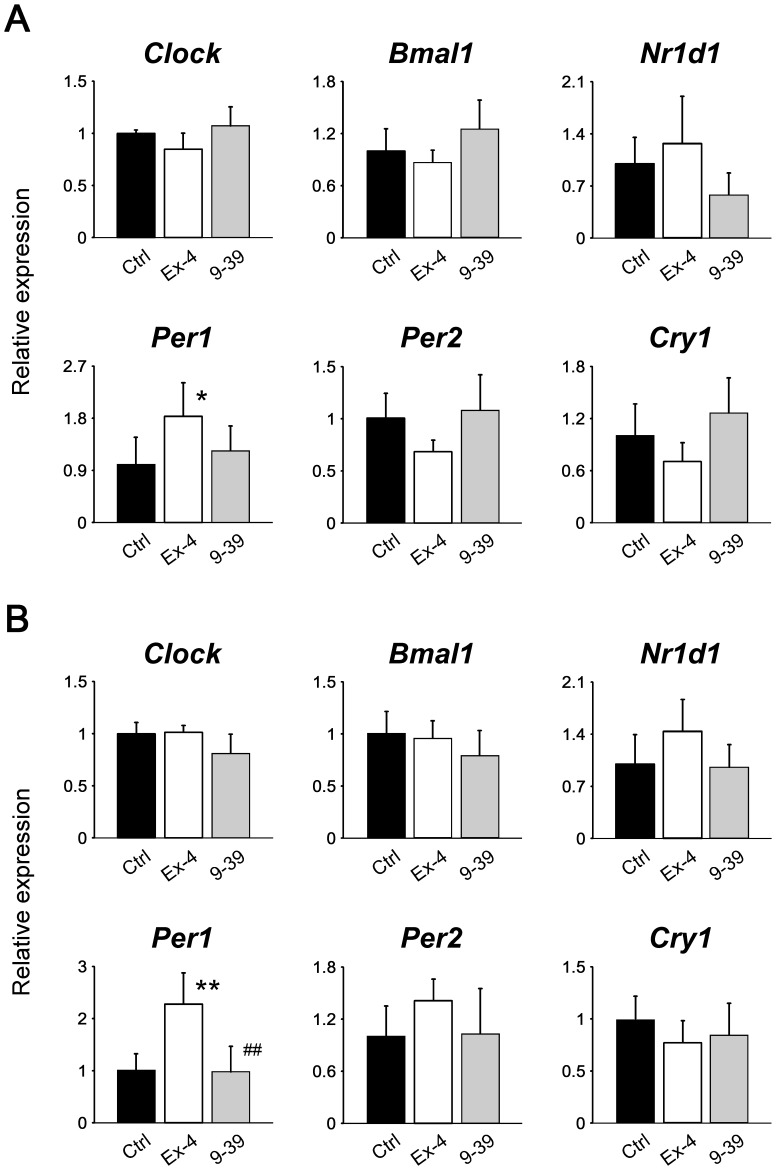
Effects of pretreatment with exendin-4 or exendin-(9–39) on refeeding-induced changes in mRNA levels of clock genes in the liver (A) and adipose tissue (B) (Experiment 3). Samples were obtained from mice treated with vehicle (Ctrl; black bars, *n* = 6), exendin-4 (Ex-4; white bars, *n* = 5), and exendin-(9–39) (9–39; gray bars, *n* = 5). The mean value of the control group was set to 1, and data represent means + SD. *, *P*<0.05, **, *P*<0.01 *vs*. the control group; ^##^, *P*<0.01 *vs*. the exendin-4 group (by one-way ANOVA followed by Bonferroni *post hoc* testing).

We further investigated whether refeeding-induced changes in mRNA levels of the clock genes are influenced by exendin-4 administration at 12 h prior to refeeding (Experiment 3). As shown in Figure 4A, pretreatment with exendin-4, but not exendin-(9–39), blunted the decreasing effect of refeeding on *Per1* in the liver (Experiment 1, Fig. 2A). Interestingly, a similar effect of exendin-4 was observed in adipose tissue (Fig. 4B). Thus, these results demonstrate that exendin-4 affected the circadian clocks in both peripheral tissues. Although food intake during the 2 h did not differ between the exendin-4 and control groups (0.027±0.003 *vs*. 0.033±0.012 g/g body weight, respectively; *P* = 0.36 by Student's *t* test), exendin-4 tended to decrease both blood glucose and serum insulin concentrations at ZT 2 (Experiment 5, Fig. S1C, S1D).

### Exendin-4 influences the food entrainment of peripheral clocks during day-time restricted feeding

**Figure 5:**
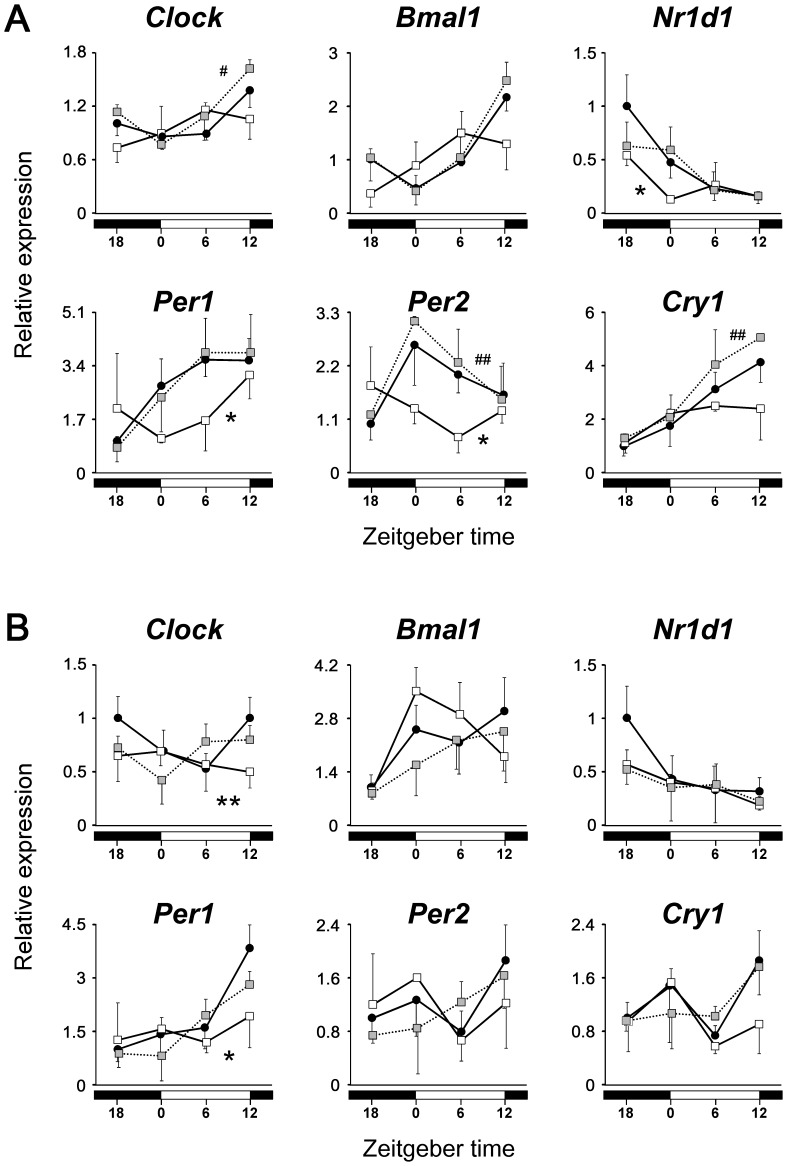
Effects of repeated administration of exendin-4 or exendin-(9–39) on daily mRNA expression profiles of clock genes in liver (A) and adipose tissue (B) (Experiment 4). Mice were fasted for 24-4 (white squares, *n* = 3 per time point), exendin-(9–39) (gray squares, *n* = 3–4 per time point), or vehicle (black circles, *n* = 5–6 per time point) at ZT 12. The mean value at ZT 18 in the control group was set to 1, and data represent means and SD. The differences of daily mRNA expression rhythms between groups were assessed by two-way ANOVA followed by Bonferroni *post hoc* testing. *, *P*<0.05, **, *P*<0.01 *vs*. the control group; ^#^, *P*<0.05, ^##^, *P*<0.01 *vs*. the exendin-4 group.

Finally, the effects of repeated administration of exendin-4 on daily rhythms of the peripheral clocks were examined. Because it is known that the administration of exendin-4 at the dosage used in this study affects feeding behavior [26], we used the protocol of time-restricted feeding in this experiment (Experiment 4, Fig. 1). As expected, food intake during the 12 h was significantly lower in the exendin-4 group than in the control group (Fig. S5), but mice treated with exendin-4, as well as those treated with exendin-(9–39) or vehicle, started eating within several minutes after being given chow on each day. Consequently, the circadian expression patterns of clock genes examined in the liver were almost reversed not only in the control group, but also in the exendin-(9–39) group (Fig. 5A, Table 1), compared with the previous results of *ad libitum*-fed mice (Table S1) [9]. However, the repeated administration of exendin-4 at ZT 12, which is the time when the active phase begins under ordinary feeding conditions [9], obviously interfered with the food-induced phase resetting of hepatic clocks (Fig. 5A, Table 1). Specifically, the treatment with exendin-4 shifted the acrophases of *Clock*, *Bmal1*, and *Per2* by 8.3, 7.6, and 8.7 h, respectively, compared with the control group. Also, in adipose tissue, exendin-4, but not exendin-(9–39), affected the rhythmicity of the expression of the clock genes examined (Fig. 5B, Table 1).

**Table 1:** Results of cosinor analysis of the clock gene expression profiles.

Clock gene	Group	*P*	Period (h)	Mesor	Amplitude	Acrophase (ZT)
Liver						
* Clock*	Control	<0.01	20.0	1.05	0.32	15.2
	Exendin-4	<0.05	26.0	0.93	0.24	6.9
	Exendin-(9–39)	<0.01	21.4	1.18	0.32	13.6
* Bmal1*	Control	<0.01	20.2	1.24	0.93	14.3
	Exendin-4	<0.01	26.0	0.95	0.64	6.7
	Exendin-(9–39)	<0.01	20.2	1.35	1.12	14.3
* Nr1d1*	Control	<0.01	26.0	0.49	0.46	18.9
	Exendin-4	n.s.				
	Exendin-(9–39)	<0.01	26.0	0.43	0.32	20.3
* Per1*	Control	<0.01	26.0	2.62	1.45	6.6
	Exendin-4	n.s.				
	Exendin-(9–39)	<0.01	26.0	2.52	1.73	7.1
* Per2*	Control	<0.01	26.0	1.82	0.71	2.4
	Exendin-4	<0.05	26.0	1.31	0.54	17.7
	Exendin-(9–39)	<0.01	26.0	2.02	0.95	1.4
* Cry1*	Control	<0.01	26.0	2.37	1.75	8.4
	Exendin-4	n.s.				
	Exendin-(9–39)	<0.01	26.0	2.90	2.13	8.4
Adipose tissue						
* Clock*	Control	<0.01	20.8	0.80	0.32	17.1
	Exendin-4	n.s.				
	Exendin-(9–39)	<0.05	26.0	0.67	0.21	10.5
* Bmal1*	Control	n.s.				
	Exendin-4	<0.01	26.0	2.26	1.29	2.7
	Exendin-(9–39)	<0.05	26.0	1.68	0.85	7.5
* Nr1d1*	Control	<0.01	26.0	0.54	0.37	18.1
	Exendin-4	n.s.				
	Exendin-(9–39)	n.s.				
* Per1*	Control	<0.01	20.0	2.15	1.51	14.1
	Exendin-4	n.s.				
	Exendin-(9–39)	<0.01	20.0	1.79	1.11	12.5
* Per2*	Control	<0.01	20.0	1.26	0.54	15.9
	Exendin-4	n.s.				
	Exendin-(9–39)	n.s.				
* Cry1*	Control	<0.05	20.0	1.28	0.52	16.5
	Exendin-4	n.s.				
	Exendin-(9–39)	<0.05	20.0	1.24	0.46	14.7

n.s., not significant.

## Discussion

The results of this study demonstrate clearly that exendin-4 affects the peripheral clocks in a manner differently than feeding. Refeeding influenced the mRNA levels of the clock genes within 2 h, whereas exendin-4 did not exhibit such effects. Interestingly, although the primary physiological stimuli for the secretion of GLP-1 are fat- and carbohydrate-rich meals, individual nutrients, including glucose and other sugars, fatty acids, essential amino acids, and dietary fiber, also can stimulate GLP-1 secretion [15], [16]. However, the phase resetting effects of glucose, sucrose, soybean oil, and casein on hepatic clocks are not detected in mice if these nutrients are given separately [27]. Collectively, these findings suggest that physiologically secreted GLP-1 is not a direct mediator of the food entrainment of peripheral clocks.

Exendin-(9–39), an N-terminally truncated peptide derivative of exendin-4, binds the GLP-1 receptor and functions as a specific GLP-1 receptor antagonist [28]. This analog has been reported to reduce postprandial insulin secretion in rats [29] and to increase plasma glucagon concentrations in humans [30]. Additionally, Waget *et al*. [31] showed in mice that exendin-(9–39) suppresses both vagus nerve activation and glucoregulation by a dipeptidyl peptidase-4 (DPP-4) inhibitor, an antidiabetic drug that increases the plasma concentration of GLP-1 [15]. Thus, these findings indicate that treatment with exendin-(9–39) can inhibit the actions of endogenous GLP-1. In this study, exendin-(9–39) did not affect the circadian clocks, suggesting that the effects of physiologically secreted GLP-1 on the clocks are minimal, if any. On the other hand, it remains possible that, similar to exendin-4, DPP-4 inhibitors influence the peripheral clocks because this class of drugs increases endogenous GLP-1, to supraphysiological levels [16]. Further studies are needed to fully clarify the effects of endogenous GLP-1 on the peripheral clocks.

Similar to GLP-1, exendin-4 has been shown in humans to stimulate insulin secretion in a glucose-dependent manner and to lower blood glucose levels [16], [18]. Glucose-stimulate insulin secretion occurs without hypoglycemia because insulin secretion drops rapidly and is nearly abolished as blood glucose concentrations fall to low levels [18]. Consistent with these properties, treatment with exendin-4 did not influence fasting concentrations of both blood glucose (Fig. S1B) and insulin (<100 pg/ml in all the mice) at ZT 12 in Experiment 5. Since blood glucose levels at the time of administration of exendin-4 (ZT 12) had decreased to relatively low values, exendin-4 did not seem to stimulate insulin secretion during the fasting period (ZT 12 to ZT 0). On the other hand, exendin-4 might transiently increase insulin secretion immediately after refeeding (at ZT 0) because the analog tended to decrease postprandial glucose levels at ZT 2 (Fig. S1C) without reduced food intake. Interestingly, Tahara et al. [24] have recently suggested that insulin mediates the feeding-induced entrainment of the liver clock. Therefore, the effects of exendin-4 on the peripheral clocks might be mediated through insulin secretion in Experiment 3, but not in Experiment 2. The discrepancy of results between Experiments 2 and 3 also suggests multiple mechanisms via which exendin-4 affects the peripheral clocks.

Our previous data suggest that leptin regulates the peripheral clocks *in vivo* [9]. Leptin has been reported to activate AMP-activated protein kinase (AMPK) in the liver and skeletal muscle [32]. AMPK regulates the circadian clock partly through degradation of the clock proteins cryptochrome 1 [33] and period 2 [34]. Additionally, peroxisome proliferator-activated receptor-γ coactivator-1α (PGC-1α), expression of which is downregulated by AMPK [35], not only regulates energy metabolism, but also stimulates the expression of clock genes *Bmal1* and *Nr1d1* [36]. Because exendin-4 and endogenous GLP-1 are also known to activate hepatic AMPK in studies using high-fat diet-fed C57BL/6J mice [37] and DPP-4-deficient rats [38], respectively, it is possible that exendin-4 influences the peripheral clocks at least partly through effects on the AMPK-PGC-1α signaling pathway.

The direct actions of GLP-1 in liver, adipose tissue, and muscle are controversial, although several studies have shown that GLP-1 and/or exendin-4 act on isolated primary rat hepatocytes or human hepatocyte cell lines [15], [37]–[39]. Interestingly, few such data have been reported in mice, and Panjwani *et al*. recently showed that both mRNA and protein levels of the GLP-1 receptor are undetectable in mouse hepatocytes [40]. Consistent with this, we did not detect any effect of exendin-4 on the mRNA expression levels of clock genes *in vitro* in liver slices from the mice (data not shown). Thus, it appears, at least in mice, that the actions of GLP-1 in liver occur through indirect mechanisms. As in the case of leptin, which acts on liver not directly, but through the sympathetic nervous system [32], GLP-1 might affect the hepatic clock via a neural mechanism. Because the 24-h fast used in this study might induce stress in mice, it was possible that the stress modified the effects of exendin-4 on the peripheral clocks through the activation of sympathetic nervous system, especially in Experiment 2.

In Experiment 4 of this study, repeated dosing of exendin-4 affected food intake, suggesting that this treatment also influenced daily profiles of both blood glucose and insulin concentrations. Blood glucose is speculated to regulate sympathetic outflow to liver via the inhibition of hypothalamic cells [41]. Therefore, the mechanisms underlying the effects of repeated administration of exendin-4 on the peripheral clocks might be fairly complex, and need to be clarified in future studies. Moreover, the physiological significance of exendin-4-induced changes in the peripheral clocks remains unclear, and additional studies are further required to elucidate whether such effects are clinically important.

In conclusion, in contrast to refeeding, a single dose of exendin-4 did not cause rapid changes in mRNA expression of the clock genes in the liver or adipose tissue. However, exendin-4 affected the clock gene mRNA levels in the liver, but not in the adipose tissue, at 12 h after administration. In addition, pretreatment with exendin-4 influenced the effect of refeeding on the circadian clocks in both peripheral tissues without affecting food intake. Furthermore, repeated doses of exendin-4 influenced both the food intake and food entrainment of circadian clocks in both tissues. These results suggest that exendin-4 modulates peripheral clocks via multiple mechanisms different from those of refeeding. Although the underlying mechanism remains to be determined, exendin-4 is a candidate agent for the treatment of disrupted peripheral clocks in patients with type 2 diabetes.

## Supporting Information

Figure S1 Effects of pretreatment with exendin-4 on blood glucose and serum insulin concentrations (Experiment 5). A, Experimental protocol. B, Mice were fasted from ZT 0 for 24 h and given exendin-4 (white squares, *n* = 9) or vehicle (black circles, *n* = 10) at ZT 12. Data represent means and SD. Blood glucose levels did not differ between the groups throughout the time points examined (*P* = 0.24 by repeated measures ANOVA). C and D, Mice were fasted from ZT 0 for 24 h, given exendin-4 (Ex-4; white bars, *n* = 4) or vehicle (Ctrl; black bars, *n* = 5) at ZT 12, and thereafter refed the regular diet from ZT 0. Samplings for determination of blood glucose levels (C) and serum insulin concentrations (D) were conducted at ZT 2. Data represent means + SD. ‡, *P*<0.1 *vs*. the control group (by Student's *t* test).
(TIF)

Figure S2 Ct values of *Rplp0* in Experiment 4. Mice were fasted for 24 h, and thereafter fed only during the light phase for 3 days. In parallel, the animals were repeatedly administered exendin-4 (white squares, *n* = 3 per time point), exendin-(9–39) (gray squares, *n* = 3–4 per time point), or vehicle (black circles, *n* = 5–6 per time point) at ZT 12. Data represent means and SD. The results of two-way ANOVA show that both the treatments and sampling time did not influence the Ct values of *Rplp0* in the liver (A) and adipose tissue (B).
(TIF)

Figure S3 Effects of refeeding, exendin-4, and exendin-(9–39) on mRNA levels of clock genes in the SCN at 2 h after the procedures (Experiment 1). Samples were obtained from control (Ctrl; black bars, *n* = 6) and refed (striped bars, *n* = 6) groups, and mice treated with exendin-4 (Ex-4; white bars, *n* = 5) and with exendin-(9–39) (9–39; gray bars, *n* = 4). The mean value of control group was set to 1, and data represent means + SD. The results of one-way ANOVA show that there are no significant differences between the groups.
(TIF)

Figure S4 Effects of exendin-4 and exendin-(9–39) on mRNA levels of clock genes in the SCN at 12 h after administration (Experiment 2). Samples were obtained from mice treated with vehicle (Ctrl; black bars, *n* = 5), exendin-4 (Ex-4; white bars, *n* = 5), and exendin-(9–39) (9–39; gray bars, *n* = 4). The mean value of the control group was set to 1, and data represent means + SD. The results of one-way ANOVA show that there are no significant differences between the groups.
(TIF)

Figure S5 Effect of repeated administration of exendin-4 on food intake (Experiment 4). Mice were fasted for 24 h, and thereafter fed only during the light phase for 3 days. In parallel, the animals were repeatedly administered exendin-4 (Ex-4; white bars, 13 mice in 3 cages) or vehicle (Ctrl; black bars, 13 mice in 3 cages) at ZT 12. Food intake was adjusted for body weight (BW), and the data represent means + SD of 3 cages. **, *P*<0.01 *vs*. the control group (by Student's *t* test).
(TIF)

Table S1 Results of cosinor analysis of the clock gene expression profiles in ad libitum-fed male C57BL/6J mice (ref. 9).
(DOCX)
