# Supplementary figures and images for: Indirect Effects of Glucagon-Like Peptide-1 Receptor Agonist Exendin-4 on the Peripheral Circadian Clocks in Mice
Source: PLoS One. 2013 Nov 15;8(11):e81119. doi: 10.1371/journal.pone.0081119 (PMC3829942; doi:10.1371/journal.pone.0081119)

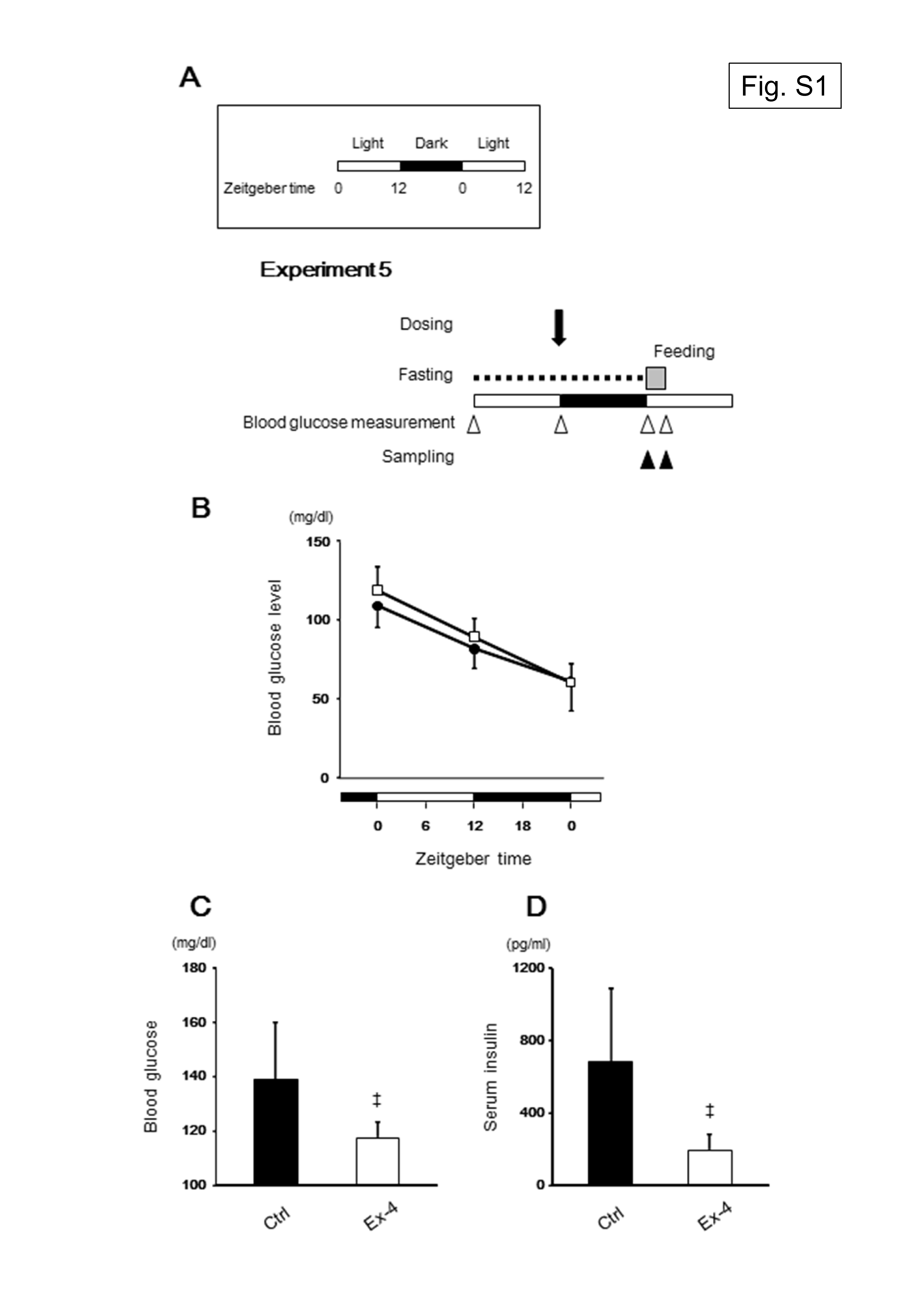

Supplement: Figure S1 — Effects of pretreatment with exendin-4 on blood glucose and serum insulin concentrations (Experiment 5). A, Experimental protocol. B, Mice were fasted from ZT 0 for 24 h and given exendin-4 (white squares, n = 9) or vehicle (black circles, n = 10) at ZT 12. Data represent means and SD. Blood glucose levels did not differ between the groups throughout the time points examined (P = 0.24 by repeated measures ANOVA). C and D, Mice were fasted from ZT 0 for 24 h, given exendin-4 (Ex-4; white bars, n = 4) or vehicle (Ctrl; black bars, n = 5) at ZT 12, and thereafter refed the regular diet from ZT 0. Samplings for determination of blood glucose levels (C) and serum insulin concentrations (D) were conducted at ZT 2. Data represent means + SD. ‡, P<0.1 vs. the control group (by Student's t test). (TIF) [file pone.0081119.s001.tif]

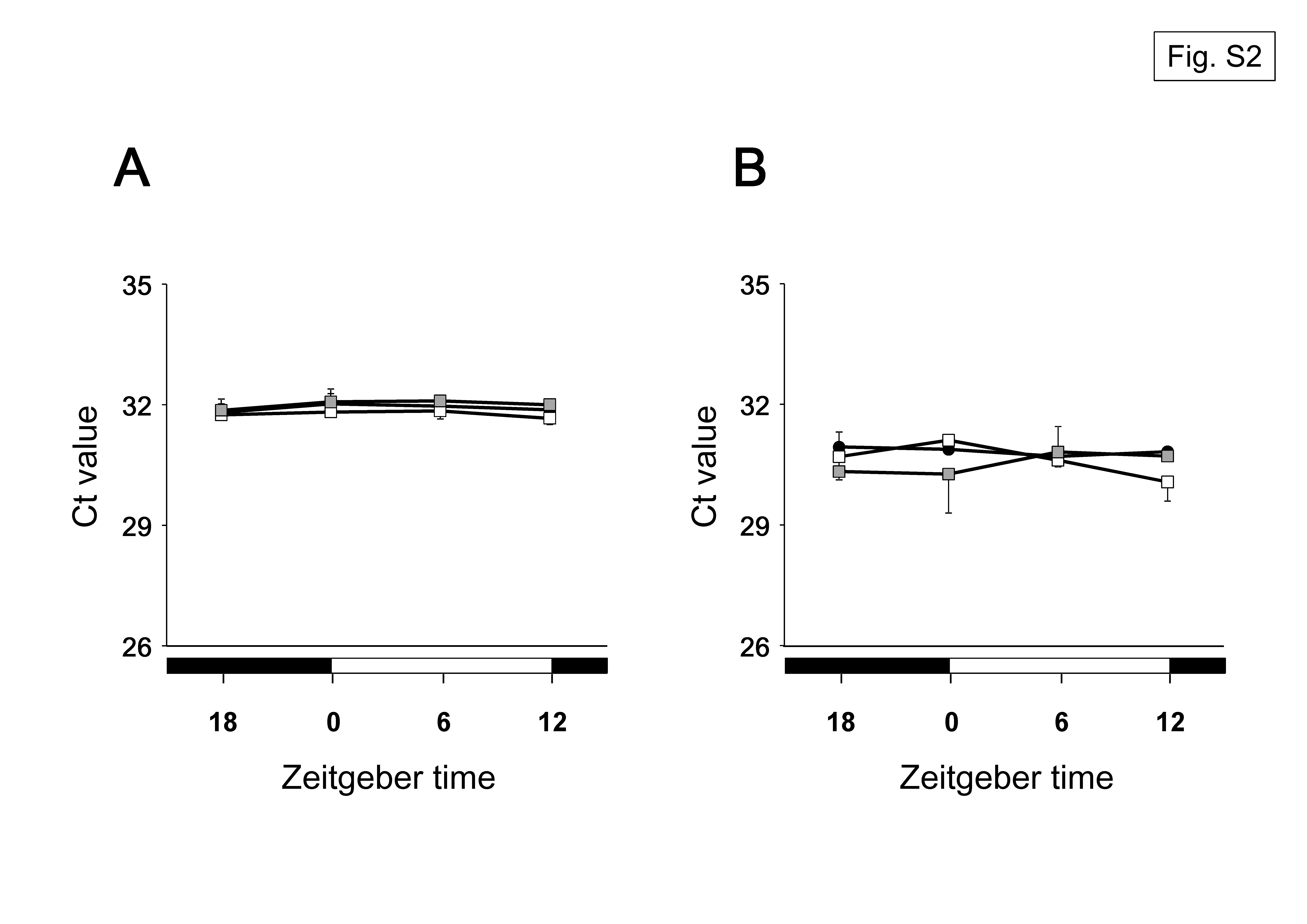

Supplement: Figure S2 — Ct values of Rplp0 in Experiment 4. Mice were fasted for 24 h, and thereafter fed only during the light phase for 3 days. In parallel, the animals were repeatedly administered exendin-4 (white squares, n = 3 per time point), exendin-(9–39) (gray squares, n = 3–4 per time point), or vehicle (black circles, n = 5–6 per time point) at ZT 12. Data represent means and SD. The results of two-way ANOVA show that both the treatments and sampling time did not influence the Ct values of Rplp0 in the liver (A) and adipose tissue (B). (TIF) [file pone.0081119.s002.tif]

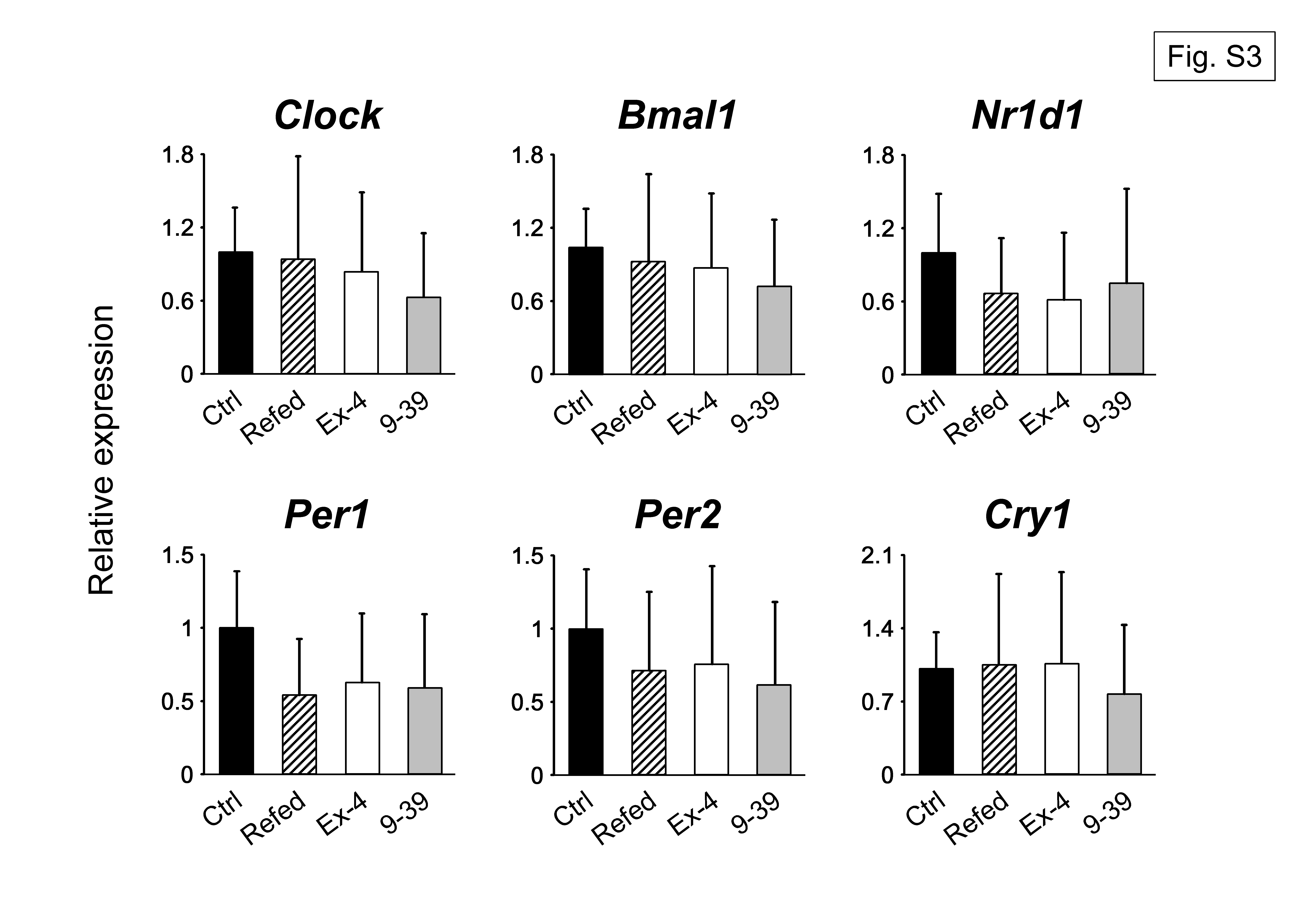

Supplement: Figure S3 — Effects of refeeding, exendin-4, and exendin-(9–39) on mRNA levels of clock genes in the SCN at 2 h after the procedures (Experiment 1). Samples were obtained from control (Ctrl; black bars, n = 6) and refed (striped bars, n = 6) groups, and mice treated with exendin-4 (Ex-4; white bars, n = 5) and with exendin-(9–39) (9–39; gray bars, n = 4). The mean value of control group was set to 1, and data represent means + SD. The results of one-way ANOVA show that there are no significant differences between the groups. (TIF) [file pone.0081119.s003.tif]

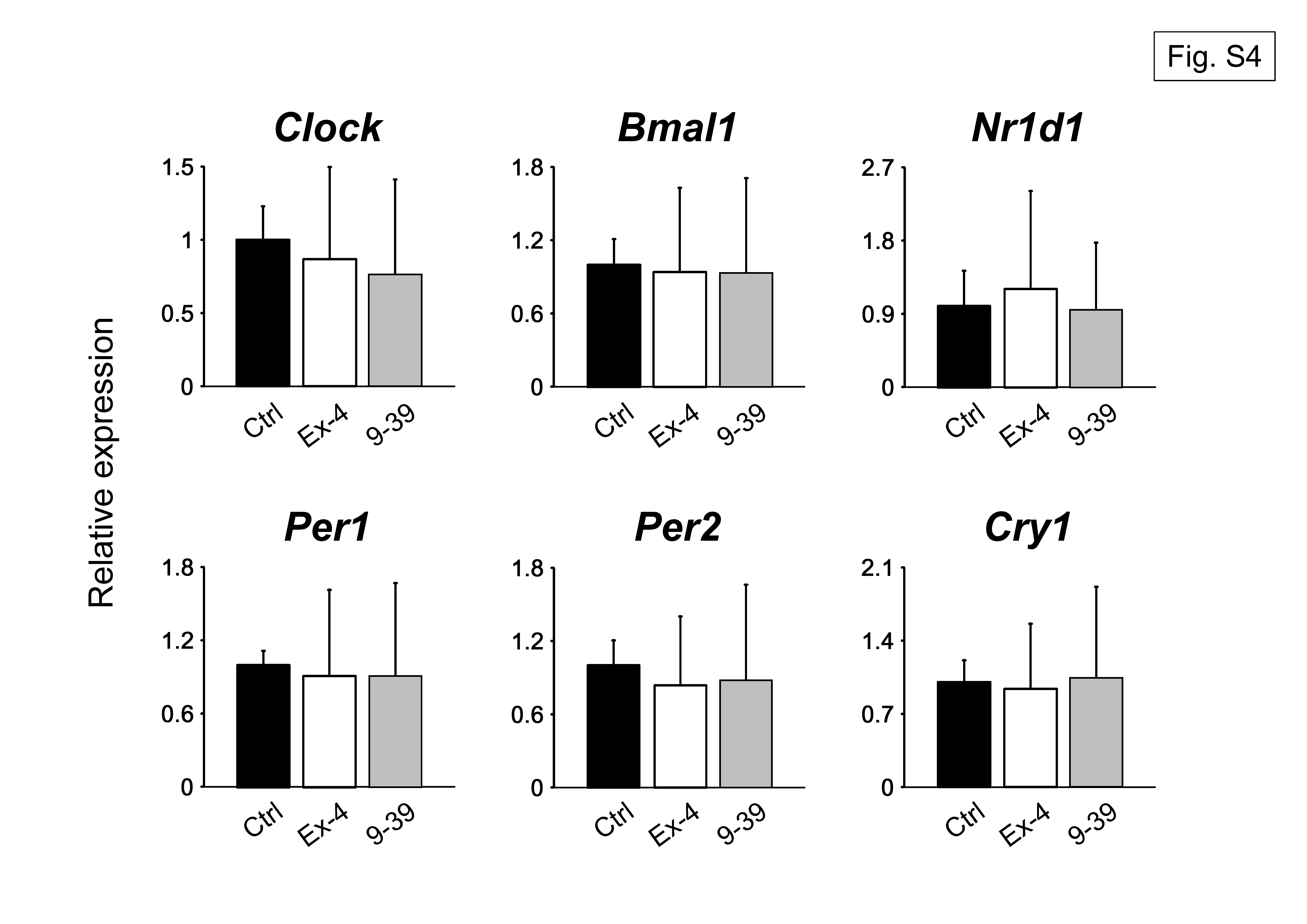

Supplement: Figure S4 — Effects of exendin-4 and exendin-(9–39) on mRNA levels of clock genes in the SCN at 12 h after administration (Experiment 2). Samples were obtained from mice treated with vehicle (Ctrl; black bars, n = 5), exendin-4 (Ex-4; white bars, n = 5), and exendin-(9–39) (9–39; gray bars, n = 4). The mean value of the control group was set to 1, and data represent means + SD. The results of one-way ANOVA show that there are no significant differences between the groups. (TIF) [file pone.0081119.s004.tif]
