# Supplementary material for: Indirect Effects of Glucagon-Like Peptide-1 Receptor Agonist Exendin-4 on the Peripheral Circadian Clocks in Mice
Source: PLoS One. 2013 Nov 15;8(11):e81119. doi: 10.1371/journal.pone.0081119 (PMC3829942; doi:10.1371/journal.pone.0081119)
Supplement: Table S1 — Results of cosinor analysis of the clock gene expression profiles in ad libitum-fed male C57BL/6J mice (ref. 9). (DOCX) [file pone.0081119.s006.docx]

| **TABLE S1.** Results of cosinor analysis of the clock gene expression profiles in *ad* *libitum*-fed male C57BL/6J mice (ref. 9) | | | | |
| --- | --- | --- | --- | --- |
|  |  |  |  |  |
|  | **Clock gene** | ***P*** | **Period (h)** | **Acrophase (ZT)** |
| Liver | |  |  |  |
|  | *Clock* | < 0.01 | 20.8 | 1.5 |
|  | *Bmal1* | < 0.01 | 26.0 | 20.9 |
|  | *Per1* | < 0.01 | 20.0 | 14.9 |
|  | *Per2* | < 0.01 | 26.0 | 14.5 |
|  | *Cry1* | < 0.01 | 26.0 | 18.4 |
| Adipose tissue | |  |  |  |
|  | *Clock* | < 0.01 | 21.0 | 3.0 |
|  | *Bmal1* | < 0.01 | 26.0 | 21.3 |
|  | *Per1* | < 0.01 | 20.0 | 13.3 |
|  | *Per2* | < 0.01 | 20.6 | 14.9 |
|  | *Cry1* | < 0.01 | 26.0 | 17.5 |
